# Supplementary material for: Symbioflor2® Escherichia coli Genotypes Enhance Ileal and Colonic Gene Expression Associated with Mucosal Defense in Gnotobiotic Mice
Source: Microorganisms. 2020 Apr 3;8(4):512. doi: 10.3390/microorganisms8040512 (PMC7232167; doi:10.3390/microorganisms8040512)
Supplement: Supplementary file 1 [file microorganisms-08-00512-s001.zip › Additional Table1 V2.0 20200326_corrected.docx]

Supplemental Table 1. Sequences of the forward and reverse primers used to detect the Symbioflor2® genotypes. A single primer pair was developed to detect G1-2, G6-7 and G8 as no specific PCR primer sets could be found to distinguish between the 3 genotypes. 3 other specific primer pairs were designed to detect the individual genotypes G3-10, G4-9 and G5.

| **Genotypes** | **Forward** | **Reverse** | **Length of amplificate** |
| --- | --- | --- | --- |
| G1-2, G6-7 and G8 | tacgtccttttcccttcgct | gaaacaggaaccgacgaaca | 95 bp |
| G4/9 | cgctatttgtaagccagttgtc | ctctgttggtgtgaatggagac | 89 bp |
| G5 | gtcgcgatgaaatcagctgt | acttccacgatgtcaccctt | 157 bp |
| G3/10 | ccatttgatcgaactgccgt | tctgaccaatccaggatcgg | 243 bp |
