# Supplementary material for: Symbioflor2® Escherichia coli Genotypes Enhance Ileal and Colonic Gene Expression Associated with Mucosal Defense in Gnotobiotic Mice
Source: Microorganisms. 2020 Apr 3;8(4):512. doi: 10.3390/microorganisms8040512 (PMC7232167; doi:10.3390/microorganisms8040512)
Supplement: Supplementary file 1 [file microorganisms-08-00512-s001.zip › sup Figure1_V2.0 20200326_corrected .pptx]

## Slide 1
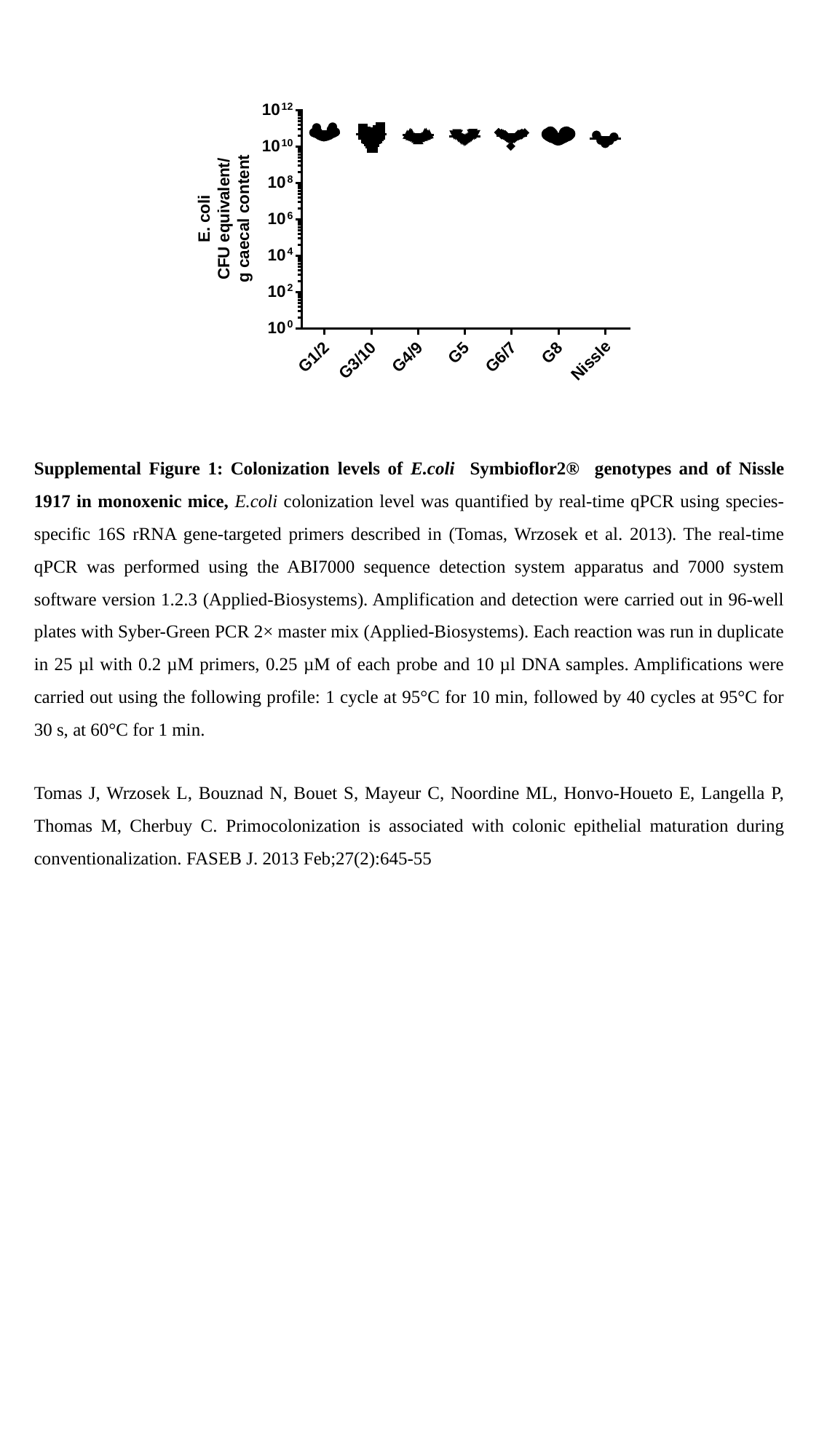

Supplemental Figure 1: Colonization levels of E.coli Symbioflor2® genotypes and of Nissle 1917 in monoxenic mice, E.coli colonization level was quantified by real-time qPCR using species-specific 16S rRNA gene-targeted primers described in (Tomas, Wrzosek et al. 2013). The real-time qPCR was performed using the ABI7000 sequence detection system apparatus and 7000 system software version 1.2.3 (Applied-Biosystems). Amplification and detection were carried out in 96-well plates with Syber-Green PCR 2× master mix (Applied-Biosystems). Each reaction was run in duplicate in 25 µl with 0.2 µM primers, 0.25 µM of each probe and 10 µl DNA samples. Amplifications were carried out using the following profile: 1 cycle at 95°C for 10 min, followed by 40 cycles at 95°C for 30 s, at 60°C for 1 min.
Tomas J, Wrzosek L, Bouznad N, Bouet S, Mayeur C, Noordine ML, Honvo-Houeto E, Langella P, Thomas M, Cherbuy C. Primocolonization is associated with colonic epithelial maturation during conventionalization. FASEB J. 2013 Feb;27(2):645-55
